# Development of a Cationic Polymeric Micellar Structure with Endosomal Escape Capability Enables Enhanced Intramuscular Transfection of mRNA-LNPs

**DOI:** 10.3390/vaccines13010025

**Published:** 2024-12-30

**Authors:** Siyuan Deng, Han Shao, Hongtao Shang, Lingjin Pang, Xiaomeng Chen, Jingyi Cao, Yi Wang, Zhao Zhao

**Affiliations:** 1 Shenzhen Neocurna Biotechnology Corporation, 12/F, Block B, Building 1, Yinxingzhijie Phase II, Longhua District, Shenzhen 518100, China; siyuandeng8023@outlook.com (S.D.); iamshaohan@outlook.com (H.S.); hongtaos@neocura.net (H.S.); lingjinpang@outlook.com (L.P.); xcm19971@gmail.com (X.C.); jingyic@neocura.net (J.C.); 2 NeoCura Bio-Medical Technology Co., Ltd., 12/F, Block B, Building 1, Yinxingzhijie Phase II, Longhua District, Shenzhen 518100, China

**Keywords:** mRNA-LNP, cationic polymeric micelle, endosomal escape, transfection efficiency

## Abstract

**Background/Objectives:** The endosomal escape of lipid nanoparticles (LNPs) is crucial for efficient mRNA-based therapeutics. Here, we present a cationic polymeric micelle (cPM) as a safe and potent co-delivery system with enhanced endosomal escape capabilities. **Methods:** We synthesized a cationic and ampholytic di-block copolymer, poly (poly (ethylene glycol)_4-5_ methacrylate_a_-*co*-hexyl methacrylate_b_)_X_-*b*-poly(butyl methacrylate_c_-*co*-dimethylaminoethyl methacrylate_d_-*co*-propyl acrylate_e_)_Y_ (p(PEG_4-5_MA_a_-*co*-HMA_b_)_X_-*b*-p(BMA_c_-*co*-DMAEMA_d_-*co*-PAA_e_)_Y_), via reversible addition–fragmentation chain transfer polymerization. The cPMs were then formulated using the synthesized polymer by the dispersion–diffusion method and characterized by dynamic light scattering (DLS) and cryo-transmission electron microscopy (CryoTEM). The membrane-destabilization activity of the cPMs was evaluated by a hemolysis assay. We performed an in vivo functional assay of firefly luciferase (Fluc) mRNA using two of the most commonly studied LNPs, SM102 LNP and Dlin-MC3-DMA LNPs. **Results:** With a particle size of 61.31 ± 0.68 nm and a zeta potential of 37.76 ± 2.18 mV, the cPMs exhibited a 2–3 times higher firefly luciferase signal at the injection site compared to the control groups without cPMs following intramuscular injection in mice, indicating the high potential of cPMs to enhance the endosomal escape efficiency of mRNA-LNPs. **Conclusions:** The developed cPM, with enhanced endosomal escape capabilities, presents a promising strategy to improve the expression efficiency of delivered mRNAs. This approach offers a novel alternative strategy with no modifications to the inherent properties of mRNA-LNPs, preventing any unforeseeable changes in formulation characteristics. Consequently, this polymer-based nanomaterial holds immense potential for clinical applications in mRNA-based vaccines.

## 1. Introduction

Messenger RNA (mRNA) has demonstrated considerable promise in the fields of infectious diseases, cancer, and protein replacement therapies [1]. Delivery systems for mRNA play a critical role in mRNA therapeutics by protecting the mRNA from degradation, enhancing successfully intracellular release and achieving effective expression [2].

Lipid nanoparticles (LNPs) have emerged as the most advanced drug delivery platforms and attracted extensive attention in mRNA delivery, particularly during the COVID-19 pandemic [3,4]. Despite the success of LNP-mRNA COVID vaccines, several challenges still remain, such as inefficient endosomal escape, limited cytosolic mRNA release, and poor targetability [5,6]. LNPs are internalized by cells through the endocytosis mechanism and subsequently transferred to early endosomes, which further mature into late endosomes and eventually fuse with lysosomes to form endolysosomes, where degradation and digestion occur [7,8,9]. It has been demonstrated that achieving endosomal escape before the late endosome or lysosome stage is crucial for efficient delivery [10,11,12]. Endosomal escape remains a critical bottleneck in the efficient delivery of nucleic acids via lipid nanoparticles (LNPs). Conventional formulations, such as MC3 and SM102 LNPs, exhibit low endosomal escape efficiencies, limiting the effectiveness of siRNA and mRNA delivery [11,13,14,15]. Endosomal escape efficiencies can also vary significantly depending on the cell type as two classes of cells are characterized by differences in the pH of their early endosomes, leading to higher complexities in endosomal escape across different cell types [11,16]. Several strategies have been explored to enhance the endosomal escape efficiency of LNPs as nucleic acid delivery systems. One approach involves using ionizable lipids or zwitterionic ionizable lipids that can become positively charged under acidic conditions in endosomes, thereby destabilizing the endosomal membrane and facilitating endosomal escape [10,13,17]. Additionally, the optimization of other LNP components, such as phospholipids [18,19], PEG lipids [20,21], or cholesterol [22,23], as well as adjustments to the LNP morphology [24], has been investigated to improve the intracellular delivery efficiency of LNP-mRNA formulations. However, it is important to realize that LNP-mRNA is a complex, multi-component formulation, where changes in one part of the system might impact the others. Focusing solely on the optimization of endosome escape efficiency may lead to unintended side effects, including increased cytotoxicity [10,25,26], adverse immune or inflammatory responses [27], or reduced stability and encapsulation efficiency [23]. Therefore, it is extremely difficult to establish a best-performing carrier through adjusting a single property of LNP formulations.

Cationic polymers have been extensively investigated as alternatives to LNPs for manufacturing delivery systems of nucleic acid therapeutics [28,29,30]. Their positive charges enable electrostatic interactions with negatively charged nucleic acids, facilitating encapsulation. These polymers become protonated at the acidified endosomal pH, which destabilizes the endosomal membrane and promotes endosomal escape [30], providing great potential for cationic polymer-based nanodelivery systems for nucleic acid therapeutics [31,32]. Commonly used cationic polymers include polyethyleneimine (PEI) [33,34,35], poly(β-amino ester) (PBAE) [36,37,38], polyaspartamide [39,40], and poly(dimethylaminoethyl methacrylate) (pDMAEMA) [41,42]. These polymers can form nanocomplexes with anionic nucleic acids directly [36,43] or can be copolymerized with hydrophilic polymers, such as polyethylene glycol (PEG), to create amphiphilic copolymers [44,45,46]. These amphiphilic copolymers self-assemble into polymeric micelles, enhancing their delivery capabilities. Despite these advantages, the cytotoxicity of cationic polymeric delivery systems remains a major challenge, hindering their broader application in nucleic acid drug delivery [47,48].

Furthermore, based on the advantages of both lipid-based and cationic polymer-based delivery systems, some studies have attempted to combine these two approaches to leverage their respective strengths. Much research has been accomplished to incorporate cationic polymers into lipid-based particles, aiming to enhance endosomal escape efficiency while ensuring safety [49,50,51]. However, the introduction of new components might affect other properties of the formulation, thus requiring further optimization for formulation development [51,52,53].

In this study, we propose a novel strategy to overcome the challenge of limited endosomal escape by co-delivering a cationic polymeric micelle (cPM), which operates independently of the LNP. The cPM, synthesized as a cationic and ampholytic di-block copolymer, is designed to disrupt endosomal membranes under acidic conditions, thus facilitating the release of mRNA into the cytoplasm. The polymer, poly (poly (ethylene glycol)_4-5_ methacrylate_a_-*co*-hexyl methacrylate_b_)_X_-*b*-poly(butyl methacrylate_c_-*co*-dimethylaminoethyl methacrylate_d_-*co*-propyl acrylate_e_)_Y_ (p(PEG_4-5_MA_a_-*co*-HMA_b_)_X_-*b*-p(BMA_c_-*co*-DMAEMA_d_-*co*-PAA_e_)_Y_), was synthesized by reversible addition–fragmentation chain transfer (RAFT) polymerization and was formulated into cPMs via the dispersion–diffusion method, resulting in nanoparticles with a size of 61.31 ± 0.68 nm, a dispersity (Ð) of 0.21 ± 0.01, and a zeta potential of 37.76 ± 2.18 mV. Hemolysis assays confirmed the endosomal escape capability of the cPMs, showing membrane disruption under endosomal pH conditions. To validate the efficacy of this approach in vivo, two widely studied LNPs, SM102 LNP and Dlin-MC3-DMA LNP, loaded with firefly luciferase (Fluc) mRNA, were co-administered with cPMs via intramuscular injection. The results demonstrated a 2–3-fold increase in the luciferase signal at the injection site compared to the controls, indicating significantly enhanced mRNA expression.

This study highlights the potential of cPM co-delivery to improve the transfection efficiency of mRNA-LNPs by enhancing endosomal escape. This innovative approach circumvents the need to modify the inherent properties of LNPs, thus avoiding unpredictable changes in formulation characteristics. These findings suggest that polymer-based nanomaterials like the cPMs developed here hold immense promise for advancing the clinical application of mRNA-based vaccines and gene therapies.

## 2. Materials and Methods

### 2.1. Materials

All chemicals were purchased from recognized commercial sources and used as received without further purification. The specific details are as follows: Polyethylene glycol 4-5 methacrylate (PEG_4-5_MA) and hexyl methacrylate (HMA) were obtained from Energy Chemical (Shanghai, China) and Bidepharm (Shanghai, China), respectively. N, N-Dimethylformamide (DMF), 4-cyano-4-(thiobenzoyl thio) pentanoic acid (CPA), deuterated methanol (CD_3_OD), deuterated chloroform (CDCl_3_), and phosphate-buffered saline powder were purchased from Sigma-Aldrich (Schnelldorf, Germany). Azodiisobutyronitrile (AIBN) was supplied by Aladdin (Shanghai, China). Butyl methacrylate (BMA) and aluminum oxide were sourced from Rhawn (Shanghai, China). Propylacrylic acid (PAA) was obtained from Hwagenpharm (Shenzhen, China). Polystyrene GPC standard was purchased from Agilent (Santa Clara, CA, USA). Methanol was obtained from Yonghuachem (Suzhou, Zhejiang, China). SM102 lipid, Dlin-MC3-DMA, high-purity cholesterol (CHO-HP), 1,2-Dioctadecanoyl-sn-glycero-3-phosphocholine (DSPC), and 1,2-dimyristoyl-rac-glycero-3-methoxypolyethylene glycol-2000 (DMG-PEG2000) were provided by AVT (Shanghai, China). Ultrafiltration tubes (MWCO 50 kDa) and Slide-A-Lyzer™ G2 dialysis cassettes (MWCO 100 kDa) were purchased from Synthware (Beijing, China) and Thermo Fisher Scientific (Waltham, MA, USA), respectively. Absolute ethyl alcohol was sourced from GHTECH (Shantou, Guangzhou, China). TritonX-100 was purchased from Solarbio (Beijing, China). Isoflurane was supplied by RWD (Shenzhen, Guangdong, China). The Quant-iT™ RiboGreen™ RNA Assay Kit, nuclease-free water, Dulbecco’s Modified Eagle Medium (DMEM), fetal bovine serum (FBS), penicillin–streptomycin, and L-glutamine were provided by Thermo Fisher Scientific (Waltham, MA, USA). Cell Counting Kit-8 (CCK8) was obtained from Dojindo (Kumamoto, Japan). D-luciferin potassium salt was sourced from Meilunbio (Dalian, Liaoning, China). Ultrapure water was produced in the laboratory using an Arium^®^ Ultrapure Water system (Sartorius, Göttingen, Germany) with a resistivity of 18.2 MΩ-cm.

### 2.2. Synthesis of Macrochain Transfer Agent (Macro-CTA): Poly(poly(ethylene glycol)_4-5_ methacrylate_a_-co-hexyl methacrylate)_b_ (p(PEG_4-5_MA_a_-co-HMA_b_))

Poly(poly(ethylene glycol)_4-5_ methacrylate_a_-*co*-hexyl methacrylate)_b_ (p(PEG_4-5_MA_a_-*co*-HMA_b_) was synthesized as a macrochain transfer agent (macro-CTA) for the subsequent synthesis of the final cationic di-block copolymer via RAFT polymerization. Briefly, the polymerization inhibitor was removed from the monomers PEG_4-5_MA and HMA by passing through columns packed with aluminum oxide. Then, the PEG_4-5_MA (3720.06 mg, 12.40 mmol) and the HMA (612.91 mg, 3.60 mmol) were dissolved in DMF at a 300 mM monomer concentration in the presence of CPA, 223.56 mg and 0.80 mmol, as a chain transfer agent (CTA) and AIBN (32.88 mg, 0.20 mmol) as an initiator. The reaction was carried out in a Schlenk tube under a nitrogen (N_2_) atmosphere at 70 °C for 18 h. The resulting product was purified using dialysis (Mw cutoff = 1000 Da) against methanol at room temperature (RT) for 3 days. Subsequently, organic solvent was eventually removed using a rotary evaporator (RV8, IKA, Staufen, Germany), yielding a purified macro-CTA for the next step.

### 2.3. Synthesis of Cationic Copolymer: Poly (poly (ethylene glycol)_4-5_ methacrylate_a_-co-hexyl methacrylate_b_)_X_-b-poly(butyl methacrylate_c_-co-dimethylaminoethyl methacrylate_d_-co-propyl acrylate_e_)_Y_ (p(PEG_4-5_MA_a_-co-HMA_b_)_X_-b-p(BMA_c_-co-DMAEMA_d_-co-PAA_e_)_Y_)

The synthesized macro-CTA p(PEG_4-5_MA_a_-*co*-HMA_b_) (208.30 mg, 0.037 mmol) and AIBN (1.50 mg, 0.0093 mmol) were dissolved in 1.47 mL of DMF. Once the macro-CTA had dissolved completely, BMA (310.00 mg, 2.18 mmol), 2-(dimethylamino)ethyl methacrylate DMAEMA (174.50 mg, 1.11 mmol), and PAA (126.70 mg, 1.11 mmol), with polymerization inhibitors removed beforehand, were added to the solution at a 3 M monomer concentration. The system’s atmosphere was purged with N_2_, and the reaction was initiated by putting the Schlenk flask into a preheated oil bath maintained at 65 °C. The reaction lasted for 22 h, and the final cationic copolymer was obtained by dialysis and rotary evaporation, as described above.

### 2.4. Proton Nuclear Magnetic Resonance Spectroscopy (^1^H-NMR)

Proton Nuclear Magnetic Resonance Spectroscopy (^1^H-NMR, AVANCE, 600 MHZ, Bruker, Billerica, MA, USA) was used to characterized the monomer compositions and chemical structures of both the synthesized macro-CTA and the cationic copolymer using CD_3_OD or CDCl_3_ as a solvent. The chemical shifts were seen from the solvent peaks at δ = 3.35 ppm for CD_3_OD and δ = 7.26 ppm for CDCl_3_.

### 2.5. Gel Permeation Chromatography (GPC)

The number average molecular weight (Mn), weight average molecular weight (Mw), and dispersity (Ð) of the synthesized macro-CTA and cationic di-block copolymer were determined using gel permeation chromatography (GPC). The analyses were carried out by using a PLgel MIXED C 7.5 mm × 300 mm 5 μm column (Agilent, Santa Clara, CA, USA) connected to an Agilent 1260 Infinity II liquid chromatography system equipped with a refractive index detector (Agilent, Santa Clara, CA, USA). Chromatographic grade DMF was selected as a mobile phase at a flow speed of 0.5 mL/min, with the column temperature set to 25 °C. Samples were dissolved in DMF at a concentration of approximately 3 mg/mL, and the injection volume was 50 μL. Calibration was achieved using a set of polystyrene standards with defined molecular weights (Mp) ranging from 162 to 89,050 Da.

### 2.6. Cationic Polymeric Micelle (cPM) Formulation

The cPMs were prepared using the dispersion–diffusion method. In detail, 30 mg of the synthesized cationic di-block copolymer was dissolved in 6 mL of methanol using a vortex mixer (RH DS025, IKA, Staufen, Germany) and then filtered through a 0.22 μm syringe filter. Subsequently, 36 mL of ultrapure water was rapidly added while stirring at 300 rpm, and the stirring speed was increased to 600 rpm for 10 min. The resulting mixture was transferred to a 50 kDa MWCO ultrafiltration tube and centrifuged at 3000 rpm for 10 min (5804R, Eppendorf, Hamburg, Germany). This process was repeated three times with additional ultrapure water to ensure the complete removal of methanol. The final solution was concentrated to 38.5 mg/mL.

### 2.7. Plasma Isolation and Protein Corona-Coated cPM (PC-cPM) Formulation

Balb/c female mice (6-8 weeks old, Beijing Vital River Laboratory Animal Technology Co., Ltd., Beijing, China) were anesthetized with isoflurane to minimize distress during blood collection. Whole blood was obtained via retro-orbital bleeding using heparinized capillary tubes and collected into EDTA pre-treated anticoagulation tubes. To isolate the plasma, the blood samples were centrifuged at 500× *g* for 5 min at 4 °C. The yellowish plasma, which forms as the supernatant after centrifugation, was carefully aspirated and transferred to sterile tubes for further analysis or storage.

cPMs (38.5 mg/mL) were incubated with undiluted plasma at a volume ratio of 1:5 at 37 °C for 2 h. Following incubation, the protein-associated cPMs were collected by centrifugation at 12,000× *g* for 15 min. The supernatant was withdrawn, and the protein corona-coated polymeric micelles (PC-cPMs) were resuspended in a buffer of pH 7.4, 0.15 M NaCl, and 1 mM EDTA at five times the volume of the supernatant. This washing step was repeated three times. Finally, the PC-cPMs were resuspended to the initial volume, assuming that the particle concentration remained constant.

### 2.8. LNP Formulation

For LNP-mRNA formulation, Fluc-mRNA was synthesized using in vitro transcription, slightly modified from a previous study [54]. The synthesized mRNA was then diluted to a concentration of 100 μg/mL using 100 mM pH 4.0 sodium citrate buffer as the aqueous phase. The organic phase was prepared by dissolving four components, including ionizable lipid (SM102 lipid or Dlin-MC3-DMA), CHO-HP, DSPC, and DMG-PEG 2000, in absolute ethyl alcohol at a molar ratio of 50:10:38.5:1.5. The lipid stock concentration was based on the nitrogen-to-phosphate ratio (N/P ratio) for the LNPs: 5.67 for the SM102 LNP and 9.94 for the MC3 LNP. The LNP-mRNA complexes were then assembled by rapidly mixing the organic phase with the mRNA aqueous phase using a microfluidics device (INano^TM^E, Micro&Nano, Shanghai, China) at a 3:1 ratio and a final flow rate of 12 mL/min. The resulting mixture was dialyzed against 10 mM pH 7.4 PBS using a Slide-A-Lyzer^TM^ G2 dialysis cassette (MWCO 100 kDa) for 18 h. The final LNP-mRNA formulation was sterilized by filtration through a 0.22 μm syringe filter, subjected to ultrafiltration and centrifugation at 1640× *g* and 4 °C, and stored at 4 °C.

### 2.9. Dynamic Light Scattering (DLS)

The particle size, PDI, and zeta potential of the cPMs, PC-cPMs, and LNPs were characterized by dynamic light scattering (DLS) at a fixed scattering angle of 175° and a temperature of 25 °C, employing a Malvern LAB (Malvern, UK). For cPMs and PC-cPMs, the measurements were conducted in water at a polymer concentration of approximately 0.39 mg/mL. For the LNPs, the DLS measurements were performed in water at an mRNA concentration of 0.05 mg/mL. Measurements were carried out in triplicate for accuracy.

### 2.10. Cryo-Transmission Electron Microscopy (CryoTEM)

The cryo-EM sample preparation of cPMs, SM102 LNP-mRNA, and MC3 LNP-mRNA was performed using a Vitrobot Mark IV system (Thermo Fisher Scientific, Waltham, MA, USA). A 3 μL aliquot of the nanoparticle suspension was placed on a pre-hydrophilized lacey carbon-coated grid (Quantifoil R1.2/1.3 Grid: Cu 300), and the excess sample was blotted away with filter paper. The grid was then rapidly blotted and plunged into liquid ethane using tweezers before being transferred to a cryo-transfer holder. Imaging was conducted using 200 kV field emission TEM (Glacios, Thermo Fisher Scientific, Waltham, MA, USA) equipped with a Falcon III direct electron detector camera.

### 2.11. RiboGreen Assay

The Quant-iT™ RiboGreen™ RNA Assay Kit was employed to determine the mRNA encapsulation efficiency (%EE) and the mRNA concentration of the LNP-mRNA following the manufacturer’s instructions. Briefly, the LNP-mRNA sample was diluted in Tris-EDTA (TE) buffer or TE buffer supplemented with 1% Triton X-100 on a black 96-well plate to achieve a final mRNA concentration of approximately 200–300 ng/mL. A series of mRNA solutions at different concentrations (0, 40, 200, 1000, and 2000 ng/mL) were prepared by gradient dilution for the standard curve. Equal volumes of 1:200-diluted RiboGreen Reagent were added to both the standards and samples. After 5 min of incubation in the dark at RT, 200 µL aliquots of each sample were transferred to a 96-well plate. Fluorescence intensity was then recorded using a microplate reader (Biotek Synergy HTX, Agilent, Santa Clara, CA, USA) with excitation and emission wavelengths of 485 nm and 528 nm, respectively. The fluorescence intensity was converted to a concentration using the standard curve generated from the mRNA standard solutions. The %EE was calculated using the following formula:

(1) $$\%EE=\frac{{Concentration}_{Triton}-{Concentration}_{No-Triton}}{{Concentration}_{Triton}}\times 100\%$$

### 2.12. Membrane-Destabilizing Activity of cPMs and PC-cPMs

#### 2.12.1. Hemolytic Analysis

Red blood cells were separated from the whole blood of Balb/c female mice (6–8 weeks old) by centrifugation at 500× *g* for 5 min at 4 °C. The red blood cells were then washed 3–4 times with PBS and finally resuspended in 100 mM PBS at pH values of 4.5, 5.5, 6.5, and 7.4 and a volume ratio of 1:50, respectively. A diluted red blood cell suspension was placed onto a 96-well plate at 180 μL per well. To monitor the endosomal escape ability of cPMs and PC-cPMs, a fixed concentration of SM102 LNPs (with a final mRNA concentration of 2.1 μg/mL; mRNA concentration is used for LNP-Fluc mRNA concentration representation throughout this study, unless otherwise specified) was mixed with a series of different concentrations of cPMs or PC-cPMs (polymer concentrations of 0, 31.25, 62.5, 125, 250, and 500 μg/mL). For each nanoparticle mixture, 20 μL was then co-incubated with red blood cells on the 96-well plates under different pH conditions for 1 h at 37 °C. PBS of a corresponding pH was used as a negative control, while 1% Triton X-100 was used as a positive control to achieve 100% lysis. After incubation, 100 μL of the supernatant was transferred to a new 96-well plate after centrifugation at 500× *g* for 5 min. Hemoglobin release was determined by measuring the absorbance at 545 nm using a microplate reader. The percentage of hemolysis was calculated based on Formula (2):

(2) $$Hemolysis\left(\%\right)=\frac{{OD}_{Sample}-{OD}_{PBS}}{{OD}_{Triton}-{OD}_{PBS}}\times 100\%$$

where the positive control represents complete lysis achieved using Triton X-100, and the negative control corresponds to red blood cells in an isotonic buffer without any treatment. This calculation provides a quantitative measure of the membrane-disrupting capability of the tested materials under various pH conditions.

#### 2.12.2. The Impact of cPMs and PC-cPM on the Stability of LNPs

The effect of cPMs and PC-cPMs on LNP-mRNA stability was determined by co-incubating SM102 LNP-mRNA (with a final mRNA concentration of 2.1 μg/mL) with a series of different concentrations of cPMs or PC-cPMs (polymer concentrations of 0, 31.25, 62.5, 125, 250, and 500 μg/mL). After 1 h of incubation at 37 °C, the free mRNA in the SM102 LNP-mRNA and cPM/PC-cPM mixture samples was measured using the Quant-iT™ RiboGreen™ RNA Assay, as described in Section 2.11.

### 2.13. In Vitro Cell Cytotoxicity Assay

HeLa cells were cultured in DMEM supplemented with 10% FBS, 1% penicillin–streptomycin, and 1% L-glutamine. Cells were cultured at 37 °C in a humidified atmosphere containing 5% CO_2_.

The cytotoxicity of the cPMs was evaluated on HeLa cells using the CCK8 assay. HeLa cells were seeded into 96-well plates at a density of 1000 cells per well and allowed to adhere overnight. The cells were then treated with various concentrations of cPMs (0, 10, 50, 100, 250, 500, and 1000 μg/mL) for 24 h. Each treatment concentration was tested in three wells to ensure reproducibility. Following treatment, 10 μL of CCK-8 reagent was added to each well, and the plates were incubated at 37 °C for an additional 2 h. The absorbance at 450 nm was measured using a microplate reader to assess cell viability. The percentage cell viability was calculated relative to the untreated control cells. This assay provided a quantitative measure of cPM-induced cytotoxicity based on the reduction in viable cell number.

### 2.14. In Vivo Animal Imaging Using the IVIS Spectrum System

All animal experiments in this study were conducted in strict accordance with the Guangdong Experimental Animal Management Regulations and Chinese Regulations of Laboratory Animals and Laboratory Animal Requirements of Environment and Housing Facilities. The mice were housed in pathogen-free cages with individual ventilation systems and kept in rooms with a controlled 12 h light/dark cycle.

The injection site was shaved 24 h prior to administration to ensure optimal imaging conditions. Female Balb/c mice (6–8 weeks old) were administered a single intramuscular injection into the quadriceps femoris muscle of one leg with either PBS, SM102 LNP-Fluc mRNA, or MC3 LNP-Fluc mRNA at a dose of 0.3 mg/kg mRNA. In an additional experimental group, mice received a combination of an intramuscular injection of LNP-mRNA (0.3 mg/kg mRNA) in one leg and cPMs (20 mg/kg) in the contralateral leg. Additionally, LNPs and cPMs were either pre-mixed or administered separately in the same leg in another set of experiments. The treatments aimed to evaluate the effects of LNP-mRNA alone and in combination with cPMs.

In the combination treatment groups, the mice received a single intramuscular injection of MC3 LNP-mRNA (0.3 mg/kg mRNA) in one leg, while varying doses of cPMs (10 mg/kg, 20 mg/kg, or 30 mg/kg) were administered in the contralateral leg. Each dose of cPMs was tested to evaluate its effect on LNP-mRNA delivery. This approach was employed to assess the impact of different cPM doses on the efficacy and safety of the LNP-mRNA formulations.

The expression of firefly luciferase protein was detected using an IVIS Spectrum System (Spectrum CT, PerkinElmer, Waltham, MA, USA) 6 h after administration. Each mouse was administrated 200 μL of D-luciferin potassium salt (15 mg/mL), which is a luciferase substrate, dissolved in PBS without magnesium and calcium via an intraperitoneal injection 12 min before imaging. Mice were anesthetized with 2% isoflurane gas for 2 min and placed in the imaging chamber. Imaging was performed using the Luminescent option for Living Image Software (version 4.4 for Windows, PerkinElmer, Waltham, MA, USA), with the exposure time set automatically. The acquired images were analyzed with Living Image Software using the Region of Interest (ROI) tool to quantify the luminescence of each mouse, expressed as total flux (photons/second).

### 2.15. Statistical Analysis

Statistical analysis was performed using GraphPad Prism 9. Data were expressed as the mean ± standard deviation of the mean as indicated. Differences between two independent groups were evaluated using an unpaired Student’s *t*-test. Statistical significance was considered at a *p*-value < 0.05.

## 3. Results

### 3.1. Characterization of Synthesized Macro-Initiator and Cationic Ampholytic Di-Block Copolymer

An ampholytic di-block copolymer composed of the first hydrophilic segment, p(PEG_4-5_MA_a_-*co*-HMA_b_), and the second hydrophobic segment, pBMA_c_-*co*-pDMAEMA_d_-*co*-pPAA_e_, was synthesized in two steps via RAFT polymerization. Table 1 presents detailed information on the two synthesized polymers, characterized by GPC and ^1^H-NMR, including Mn, Mw, Ð, and the monomer ratio.

**Figure 1:**
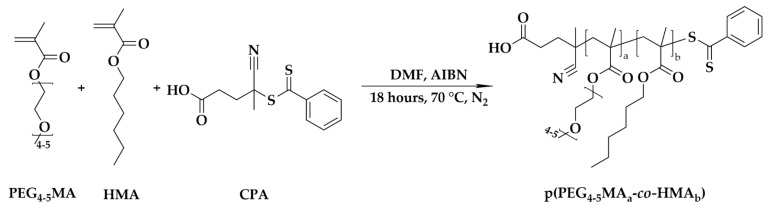
Synthesis of p(PEG_4-5_MA)_a_-*co*-pHMA_b_ as a macro-CTA via RAFT polymerization.

CPA and AIBN, a well-established chain transfer agent and initiator pair, were utilized for the RAFT polymerization of the first block of the di-block copolymer composed of pPEG_4-5_MA and pHMA. The detailed synthetic route is illustrated in Figure 1. The resulting macro-CTA p(PEG_4-5_MA)_a_-*co*-pHMA_b_ exhibited a Ð of 1.1 with a narrow and symmetric peak in GPC, which confirmed the controlled copolymerization and unimodal molecular weight distribution. The monomer ratio of PEG_4-5_MA to HMA was calculated by comparing the integration ratio of the characteristic methylene group of PEG_4-5_MA at 4.14 ppm with the methylene group of HMA at 3.98 ppm (Figure S1). The results indicated that 85.7% of the PEG_4-5_MA monomer was integrated into p(PEG_4-5_MA)_a_-*co*-pHMA_b_, which is close to the 78% present in the feed.

**Figure 2:**
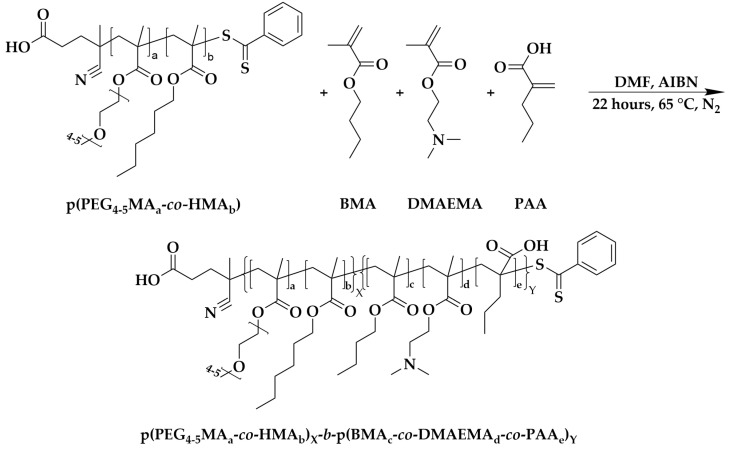
Synthesis of {poly(poly(ethylene glycol)_4-5_ methacrylate)_a_-*co*-poly(hexyl methacrylate)_b_}_X_-*b*-{poly(butyl methacrylate)_c_-*co*-poly(dimethylaminoethyl methacrylate)_d_-*co*-poly(propyl acrylate)_e_}_Y_ ({p(PEG_4-5_MA)_a_-*co*-pHMA_b_}_X_-*b*-{pBMA_c_-*co*-pDMAEMA_d_-*co*-pPAA_e_}_Y_) via RAFT polymerization.

Next, the second segment of the di-block ampholytic copolymer, composed of BMA, DMAEMA, and PAA, was synthesized through RAFT polymerization. This process utilized the resulting p(PEG_4-5_MA)_a_-*co*-pHMA_b_ as a macro-CTA and AIBN as an initiator, following the RAFT polymerization method illustrated in Figure 2. The final cationic di-block amphophilic copolymer, produced with a yield of 70.77%, displayed Mn, Mw, and PDI values of 39.8 kDa, 64.6 kDa, and 1.4, respectively. The chemical structure of the copolymer was determined by ^1^H-NMR spectroscopy (Figure S2). The peak at 2.27 ppm was attributed to the terminal methyl groups of DMAEMA. The peak at 3.92 ppm corresponded to the methylene group next to the ester bonds of HMA from the first segment and BMA from the second segment. The 4.06 ppm peak was due to the methylene group next to the ester bonds of the PEG and DMAEMA segments. The characteristic cross-peak at (0.86–1.02) ppm was attributed to the ending methyl groups of HMA, BMA, and PAA. The second segment of the copolymer was composed of 50.0% BMA, 36.2% DMAEMA, and 13.8% PAA, as determined through ^1^H NMR and GPC analyses.

### 3.2. Characterization of LNPs and cPMs

Two widely used and well-validated LNPs for nucleic acid delivery, SM102 LNP and MC3 LNP, were selected and formulated using microfluidic technology as benchmarks to assess whether cationic polymeric micelles (cPMs) could enhance the in vivo expression efficiency of LNPs. The particle size, PDI, zeta potential, and mRNA encapsulation efficiency of the LNPs, i.e., %EE, were characterized and are summarized in Table 2. Both of the Fluc mRNA-loaded LNPs exhibited a spatially uniform distribution with an average particle size of approximately 80–90 nm, as indicated by the low PDI values. Both of the LNPs were slightly positively charged with a zeta potential of approximately 10 mV, induced by the cationic lipids.

**Figure 3:**
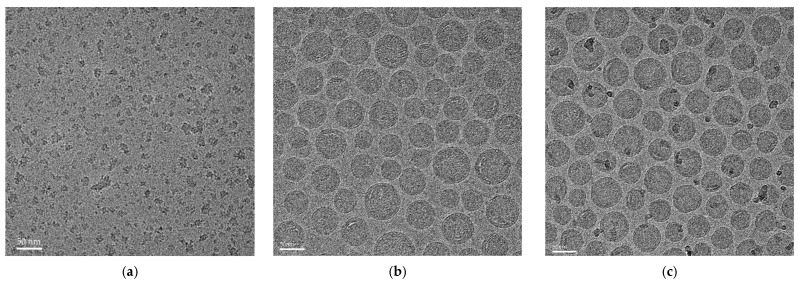
Cryo-TEM images of (**a**) cPMs; (**b**) SM102 LNP-mRNA; and (**c**) MC3 LNP-mRNA (scale bar is 50 nm for all the images).

The cationic polymeric micelles were further formulated using the dispersion–diffusion method. Briefly, the synthesized cationic di-block copolymer was completely dissolved in methanol and then transferred to water to induce self-assembly driven by hydrophilic and hydrophobic interactions, resulting in the formation of micellar structures, supported by the DLS and cryo-TEM results. The DLS measurements revealed a particle size of 61.31 ± 0.68 nm, a PDI of 0.21 ± 0.01, and a zeta potential of 37.76 ± 2.18 mV. To assess the morphology, cryo-TEM imaging was performed. As shown in Figure 3, the cPMs displayed a nearly spherical shape with a rough surface. Cryo-TEM analysis indicated a particle size distribution of approximately 30–40 nm, which is smaller than that measured by DLS. This minor discrepancy arises because, in the DLS measurements, cPMs in solution may form aggregates due to their positive charge. In contrast, cryo-TEM captures the sample in a dry state with a stronger emphasis on smaller components, providing a precise readout that is closer to the true particle size of the cPMs. SM102 LNP-mRNA and MC3 LNP-mRNA were also analyzed via cryo-TEM, as shown in Figure 3b,c, revealing spherical nanoparticles with a uniform size distribution, characteristic of well-formed lipid nanoparticles.

### 3.3. The Impact of cPMs and PC-cPMs on the Structural Stability and Endosomal Escape Capabilities of LNPs

To more accurately replicate the in vivo performance of cPMs, including their association with LNPs and subsequent interactions with the endosomal membrane post-endocytosis, cPMs were co-incubated with mice blood plasma to simulate the formation of a protein corona during their circulation in the bloodstream. After two hours of co-incubation at 37 °C, the cPMs were collected by centrifugation. DLS analysis showed an approximate increase of 200 nm in particle size, accompanied by a shift in the zeta potential from positive to negative, as shown in Table 2. These results indicate protein adsorption on the cPM surface, forming a protein corona around the cPMs during co-incubation with plasma.

While there is no agreed-upon method developed to detect and quantify endosomal escape [5], we followed a typical pH-dependent hemolysis assay, which has been widely used as a surrogate strategy to quantify the endosomal escape potential of polymers [55]. By measuring the extent of hemoglobin release resulting from the disruption of red blood cell membranes upon exposure to cPMs or protein corona-coated cPMs (PC-cPM), this assay was used to assess the membrane-destabilization activity of both cPMs and PC-cPMs. Furthermore, we compared the hemolysis effects of cPMs or PC-cPMs combined with LNP-mRNA formulations against those of LNP-mRNA alone. This indirect approach probes the potential of cPMs and PC-cPMs to enhance the endosomal escape efficiency of mRNA-LNPs [56,57]. SM102 LNP-Fluc mRNA, both alone and in cooperation with cPMs or PC-cPMs, was evaluated under different pH conditions through hemolysis assays using red blood cells. Four distinct pH values—7.4, 6.5, 5.5, and 4.5—were chosen to simulate physiological conditions, late endosomes, early endosomes, and lysosomes, respectively.

**Figure 4:**
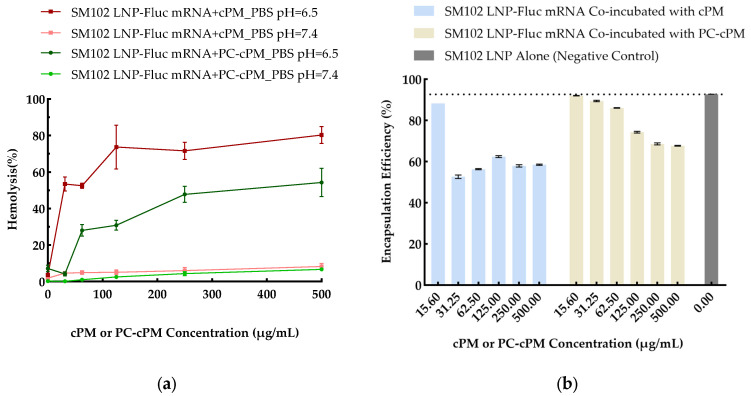
Membrane-destabilizing activity of cPMs and PC-cPMs: (**a**). Evaluation of membrane-destabilizing activity of cPMs and PC-cPMs (0–500 μg/mL) in combination with SM102 LNPs (2.1 μg/mL) by hemolysis assay under early endosomal (pH 6.5) and physiological (pH 7.4) conditions (**b**). Effects of cPMs and PC-cPMs (0–500 μg/mL) on encapsulation efficiency of SM102 LNPs (2.1 μg/mL), with implications for formulation stability.

As shown in Figure S3a, SM102 LNPs alone showed minimal hemolysis at pH 7.4 and pH 6.5 across all the tested concentrations. Unsurprisingly, complete hemolysis was observed at low pH levels. Even at the lowest concentration of 1.09 μg/mL, SM102 LNP-Fluc mRNA exhibited approximately 80% hemolysis at pH 4.5 and 5.5. Subsequently, cPMs and PC-cPMs at different concentrations (0–500 μg/mL) were separately co-incubated with SM102 LNP-Fluc mRNA (2.1 μg/mL) and red blood cells under varying pH conditions. Consistent with these observations, nearly 100% hemolysis was achieved at pH 4.5 and 5.5 (Figure S3b). Strikingly, at pH 6.5, which is typical of early endosomes, the inclusion of cPMs or PC-cPMs significantly increased the hemolysis rate from approximately 5% (with SM102 LNP-Fluc mRNA alone) to about 80% for cPMs and 50% for PC-cPMs at the highest concentration (Figure 4a). These results suggest that cPMs can enhance the membrane-disruption capability of SM102 LNP-Fluc mRNA under conditions resembling those of early endosomes, thus facilitating endosomal escape. Although the PC-cPMs consistently demonstrated lower hemolytic efficiency compared to the cPMs across all concentrations, they substantially promoted the overall hemolytic efficiency of the formulation to above 50% when the PC-cPM concentration exceeded 250 μg/mL.

Considering the biomimetic lipid-based structures of LNPs, we next asked whether the cPMs affected the stability of LNPs via their membrane-disruption ability. SM102 LNP-Fluc mRNA was incubated with cPMs at different concentrations from 0 to 500 μg/mL, and the %EE was measured by dividing the amount of encapsulated mRNA by the total amount of mRNA in the nanoparticle mixture (Figure 4b). Specifically, when the cPM concentration exceeded 31.25 μg/mL, the %EE of SM102 LNP-Fluc mRNA dropped dramatically to around 50%. Compared to the cPMs, the %EE of SM102 LNP-Fluc mRNA remained 20% to 30% higher when co-incubated with PC-cPMs at the same concentration. Specifically, the encapsulation efficiency of SM102 LNP-Fluc mRNA increased from approximately 55% with uncoated cPMs to about 85% with PC-cPMs at a concentration of 62.5 μg/mL. These data indicate that cPMs have the ability to destabilize the SM102 LNP membrane, leading to Fluc mRNA leakage and possibly affecting its expression in vivo. More importantly, the formation of protein corona was found to reduce the effect of cPMs on the stability of SM102.

### 3.4. Cytocompatibility of cPMs

**Figure 5:**
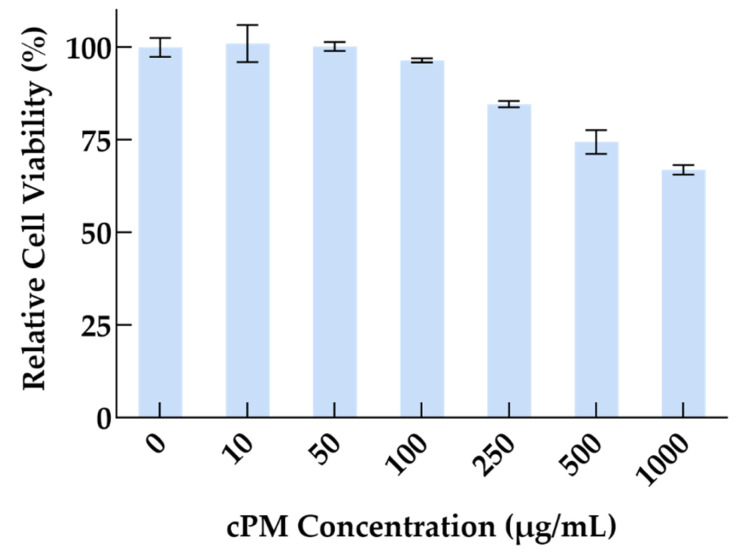
Cytotoxicity of cPMs after incubation for 24 h.

The cytocompatibility of the developed cPMs was assessed using the CCK8 assay with HeLa cells. After 24 h of incubation, no significant cytotoxicity was observed when the concentration of the cPMs was below 100 μg/mL (Figure 5). A mild cytotoxic effect of the cPMs was detected in the concentration range between 250 and 1000 μg/mL, reducing the cell viability to 75%.

### 3.5. Bioluminescence Imaging of LNP-Fluc mRNA and cPMs Following Intramuscular Administration

To investigate whether cPMs can enhance the intracellular delivery efficiency of LNPs, cPMs (20 mg/kg) and MC3 LNP-Fluc mRNA (0.3 mg/kg, mRNA) were administered to mice through different procedures.

**Figure 6:**
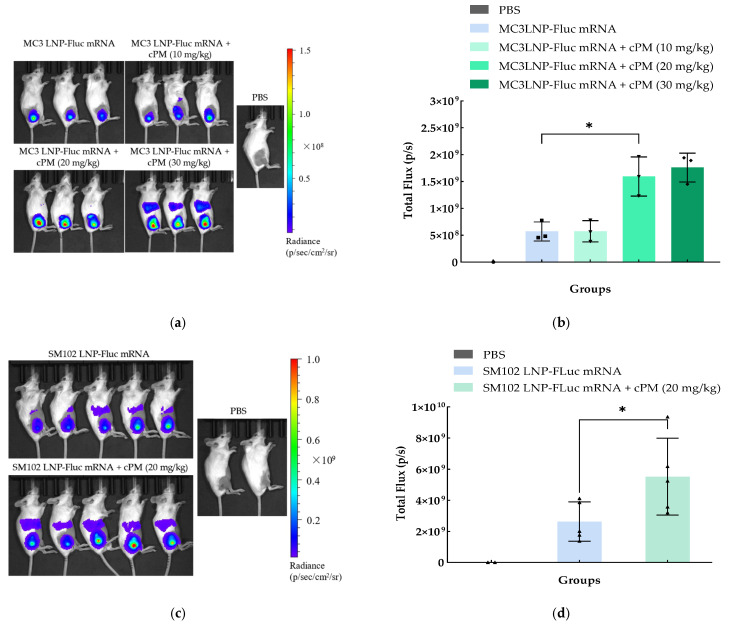
Evaluation of intramuscular administration of MC3 LNP-Fluc mRNA (**a**,**b**) or SM102 LNP-Fluc mRNA and (**c**,**d**) in vivo expression with separate cPM administration in contralateral legs. (**a**) Representative images of luciferase expression at the MC3 LNP-Fluc mRNA injection site in the whole body 6 h after administration. (**b**) Total flux (p/s) of luciferase activity at the MC3 LNP-Fluc mRNA injection site calculated using Living imaging software; an unpaired Student’s *t*-test was performed between the group that received only MC3 LNP-Fluc and the group that received MC3 LNP-Fluc mRNA and cPMs (20 mg/kg) in contralateral legs; * *p <* 0.05. Data are presented as mean ± standard deviation; *n* = 3 per experiment group. (**c**) Representative images of luciferase expression at the SM102 LNP-Fluc mRNA injection site in the whole body 6 h after administration. (**d**) Total flux (p/s) of luciferase activity at the SM102 LNP-Fluc mRNA injection site calculated using Living imaging software; an unpaired Student’s *t*-test was performed between the group that received only SM02 LNP-Fluc and the group that received SM102 LNP-Fluc mRNA and cPMs (20 mg/kg) in contralateral legs; * *p <* 0.05. Data are presented as mean ± standard deviation; *n* = 5 per experiment group.

Initially, cPMs were pre-mixed with MC3 LNP-Fluc mRNA and then injected into the mice intramuscularly. However, six hours post-injection, no bioluminescence signal was detected, while the total flux reading at the injection site was comparable to that of the negative control group, which only received PBS injections (Figure S4). This observation led us to hypothesize that the membrane-disruptive properties of cPMs might compromise the stability of the LNP formulation in the process of pre-mixing. To better promote LNP-mRNA expression while omitting the pre-mixture step before administering the intramuscular injections, two separate injection strategies were attempted. In the first approach, MC3 LNP-Fluc mRNA and cPMs were administered separately into the same quadriceps femoris muscle of the mice. At six hours post-injection, the mice who received two separate injections of MC3 LNP-Fluc mRNA and cPMs showed an approximately three-fold decrease in bioluminescence signals compared to the group that received a single injection of MC3 LNP-Fluc mRNA alone (Figure S5). In the second approach, cPMs and MC3 LNP-Fluc mRNA were administered intramuscularly into different legs of the mice. Interestingly, there was a robust, 2–3-fold increase in the signal intensity of the bioluminescence signal from the mice who received separate cPM administration in the contralateral leg than that in the control group injected with LNP-Fluc mRNA alone at six hours post-injection (Figure 6a,b). To further validate these findings, we repeated these in vivo experiments with SM102 LNP-Fluc mRNA. Similarly, the mice who received separate injections of SM102 LNP-Fluc mRNA and cPMs exhibited a 2-fold greater bioluminescence signal than that in the group receiving SM102 LNP-Fluc mRNA alone (Figure 6c,d), confirming the reproducibility of the results.

To this end, we further investigated the dose effect of cPMs in promoting mRNA-LNP delivery. As shown in Figure 6a,b, a comparative analysis of MC3 LNP Fluc mRNA delivery was performed with cPMs at three doses of 10 mg/kg, 20 mg/kg, and 30 mg/kg. At the lowest dose of 10 mg/kg, cPMs produced a comparable bioluminescent signal to the control mice injected with MC3 LNP-Fluc mRNA only. However, we found a marked increase in the Fluc signal with increasing doses of cPMs from 10 mg/kg to 20 mg/kg, whereas the luciferase signal remained constant at the highest dose tested (30 mg/kg). However, at 30 mg/kg, the liver signal increased significantly, which may indicate enhanced hepatic accumulation or a preferential biodistribution of cPMs at higher concentrations, potentially driven by polycationic interactions with liver-resident cells or other hepatic components.

The body weight of the mice was monitored both prior to and following administration. As illustrated in Figure S6a,b, there was no significant weight loss observed across all groups within 24 h post-administration. Less than 10% total body weight loss was observed over the 48 h post-administration, indicating no severe adverse effects related to the treatment.

## 4. Discussion

In this study, we aimed to address the persistent challenge of inefficient endosomal escape and suboptimal cytosolic mRNA release, which continue to limit the efficacy of LNP-mRNA delivery systems. Traditional strategies, such as the use of ionizable lipids or the optimization of other LNP components, have made strides in improving endosomal escape; however, they often introduce trade-offs that complicate the overall formulation [1,2,3]. Furthermore, cationic polymers such as PEI, PBAE, and pDMAEMA have been explored as promising alternatives to LNPs for nucleic acid delivery [35,36,41,42]. These polymers possess positive charges, which not only facilitates the encapsulation of negatively charged nucleic acids but also enables them to become protonated at acidic pH levels, thereby disrupting endosomal membranes. Despite the improvement in endosomal escape capability, cationic polymers exhibit lower delivery efficiency and stronger cytotoxicity compared to LNPs [12]. Here, we proposed a novel strategy that utilizes the co-delivery of endosome-disrupting cPMs alongside an LNP-mRNA formulation to enhance endosomal escape, thereby increasing the transfection efficiency of mRNA. The major advantage of our approach lies in its use of two distinct formulations; this approach allows us to leverage their individual strengths and avoid the negative impacts that might be produced from combining multiple components within a single formulation.

The developed cationic di-block copolymer, composed of both hydrophilic and hydrophobic segments, enables the formation of polymeric micelles through amphiphilic interactions. The hydrophilic segment comprises PEG_4-5_MA and HMA. Relative to linear PEG, we selected brush-style PEG due to its enhanced potential for cellular uptake and interaction, as well as its ability to provide a more favorable microenvironment for targeted delivery [8,9,10]. Additionally, we also incorporated hydrophobic HMA for its role in promoting endocytosis, thus increasing the cellular uptake of PEG-coated nanoparticles [58,59,60]. The subsequent hydrophobic segment, composed of BMA, DMAEMA, and PAA, was copolymerized using p(PEG_4-5_MA)_a_-*co*-pHMA_b_ as a macro-CTA. While DMAEMA enhances the copolymer’s endosomal escape capability through its positive charge, this effect is counterbalanced by its associated cytotoxicity [61,62]. Copolymerization with pH-sensitive anionic PAA enhances the biocompatibility and stability of the polymeric micelles by neutralizing the positive charge of DMAEMA at a physiological pH [63]. And in the acidic endosomal environment, PAA becomes more hydrophobic by losing its charge, while DMAEMA increases its positive charge by protonation. These pH-dependent protonation behaviors may facilitate endosomal membrane disruption [64,65,66]. BMA was selected to further increase the hydrophobicity of the copolymer, improving the performance of the secondary hydrophobic segment [64]. Since cationic copolymers with similar structures have been utilized in other studies for nucleic acid delivery and have demonstrated significant endosomal escape capabilities [64,65,66,67], here, we present a novel application of a cationic copolymer as an mRNA adjuvant when administered in two separate nanoparticle systems.

**Figure 7:**
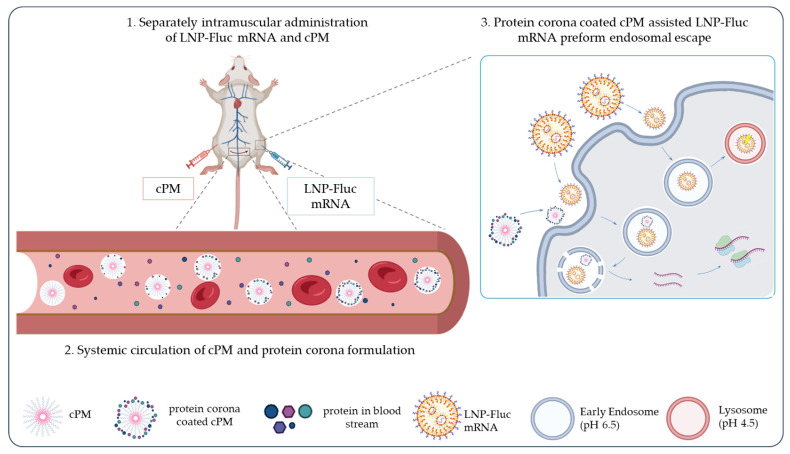
Schematic diagram of cPM-assisted enhancement of LNP-Fluc mRNA expression: 1. cPMs and LNP-Fluc mRNA are separately administered into the contralateral legs of mice (i.m.); 2. cPMs enter into the bloodstream through capillaries, where they are bound by the protein corona; and 3. upon their arrival at the LNP injection site on the contralateral leg, PC-cPMs promote endosomal escape via co-endocytosis with the LNPs, leading to the improved expression efficiency of LNP-Fluc mRNA.

Cationic copolymers with similar structures have been reported to enhance the intracellular delivery efficiency of siRNA and mRNA due to their improved endosomal escape capacity [67,68,69,70]. However, this exploratory research provides empirical evidence for such cationic polymeric micellar structures to enhance the endosomal escape efficiency of another mRNA delivery vector via intramuscular administration, indicating great promise for clinical applications in therapeutic vaccines. Furthermore, we demonstrated that the cPMs can affect the stability of the LNP formulations in vitro through their membrane-disruptive effects. We tested whether the separate administration of cPMs and LNPs in contralateral legs was a feasible strategy to promote LNP-mRNA delivery while keeping the LNPs relatively stable. We observed a marked increase in the expression of Fluc-mRNA in vivo. As shown in a number of nanoparticle studies [71,72,73], cPMs may enter the bloodstream through capillaries at the injection site after intramuscular administration, from which point they circulate throughout the body and then potentially reach the LNP injection site in the contralateral leg. Numerous previous studies have also shown that nanoparticles in the bloodstream can interact with proteins to form a protein corona, which may alter the surface characteristics of the nanoparticles [74,75,76]. In particular, positively charged nanoparticles are more prone to protein adsorption in circulation [77,78]. As demonstrated in the in vitro assay using PC-cPMs, the reduced impact of cPMs on LNP stability observed indicated the shielding role of the protein corona on the cPMs. These data are in good agreement with other studies [79,80,81] carried out on other polymeric nanoparticles. As polymeric micelles are known to perform endocytosis as their main route for cell internalization [82], we propose that PC-cPMs may undergo endocytosis together with LNP-mRNA, thus enhancing endosomal escape and improving the expression efficiency of the delivered mRNA. Here, we provide a schematic representation of cPM-assisted LNP-mRNA delivery, as illustrated in Figure 7.

Overall, our work represents a pioneering conceptual study for the design and synthesis of cationic polymeric micelles, as well as the underlying mechanism of protein corona-mediated cationic polymeric micelle-assisted LNP-mRNA delivery. This study is quite preliminary and requires further investigation. The key areas of focus for future research include the optimization of polymer targeting and dosage ratios to ensure better efficacy. Further adjustments to balance the cationic and amphiphilic content of the di-block copolymer are needed to minimize its impact on LNP-mRNA stability while maximizing endosomal escape efficiency. Also, given the repeated unit in cPMs, higher positive charges may cause cell membrane damage and higher cytotoxicity. To face the PEG dilemma, further exploration is crucial to balance delivery efficiency with safety. Aside from charge, the distinct hydrophilic and hydrophobic properties of the polymer can also affect the biodistribution of nanoparticles [83]. To overcome the limitations of conventional delivery, the optimization of each chemical entity could help improve targeted delivery. Future investigations will also focus on conducting comprehensive in vivo evaluations to rigorously assess the long-term safety and biocompatibility of these co-formulations, thereby advancing their potential for clinical applications. Further research has to be carried out to learn more about how cPMs enter the bloodstream, circulate, and reach the LNP-mRNA sites and to enhance the expression of the delivered mRNAs. Lastly, while this study utilized the firefly luciferase model protein, validation with other proteins of practical significance is necessary for broader applicability.

## 5. Conclusions

This study presents a novel approach to enhancing mRNA delivery efficiency by co-delivering cPMs alongside LNPs, aiming to address the critical challenge of endosomal escape. The synthesized cPMs, characterized by their distinct hydrophilic and hydrophobic segments, demonstrated effective membrane disruption under acidic conditions, simulating the endosomal environment.

Our findings reveal that the intramuscular administration of cPMs in conjunction with LNPs significantly increased firefly luciferase expression at the injection site, achieving up to a 2–3-fold higher signal compared to the control groups. This result suggests that the cPMs substantially enhance the endosomal escape efficiency of LNP-mRNA complexes, leading to improved transfection efficiency.

The developed cPMs offer a strategic advantage by improving endosomal escape without altering the intrinsic properties of LNPs. This approach provides a promising alternative for optimizing mRNA delivery systems, holding great potential for a plethora of clinical applications, such as mRNA-based vaccines and gene therapies. Future research directions include the formulation optimization of the cationic polymeric micelles as well as the therapeutic molecules that work for this delivery system for improved clinical utility.

## 6. Patents

Patent is titled [Translated] Cationic polymer, preparation method and application thereof, and mRNA delivery system (202410685294X) were submitted to the China National Intellectual Property Administration on 30 May 2024.

## Figures and Tables

**Table 1:** Mn, Mw, Ð, and monomer ratio of p(PEG_4-5_MA)_a_-*co*-pHMA_b_ and p(PEG_4-5_MA)_a_-*co*-pHMA_b_}_X_-*b*-{pBMA_c_-*co*-pDMAEMA_d_-*co*-pPAA_e_}_Y_.

Polymer	Mn ^1^ (kDa)	Mw ^1^ (kDa)	Ð ^1^	1st Block		2nd Block		
				% PEG_4-5_MA ^2^	% HMA ^2^	% BMA ^2^	% DMAEMA ^2^	% PAA ^2^
p(PEG_4-5_MA_a_-*co*-HMA_b_)	7.3	8.1	1.1	85.7	14.3	/	/	/
p(PEG_4-5_MA_a_-*co*-HMA_b_)_X_-*b*-p(BMA_c_-*co*-DMAEMA_d_-*co*-PAA_e_)_Y_	39.8	64.3	1.6	85.7	14.3	50.0	36.2	13.8

^1^ As assessed by GPC; ^2^ as assessed by ^1^H-NMR.

**Table 2:** Particle size, PDI, and zeta potential of nanoparticles.

Nanoparticle	Size (nm)		PDI		Zeta (mV)		EE (%)
	Ave.	Sta.Dev.	Ave.	Sta.Dev.	Ave.	Sta.Dev.	
SM102 LNP	90.11	1.3	0.14	0.09	10.15	0.28	91
MC3 LNP	83.63	0.19	0.15	0.03	10.77	0.80	93
cPM	61.31	0.68	0.21	0.01	37.76	2.18	/
PC-cPM	265.70	15.31	0.25	0.05	−13.67	1.46	/

## Data Availability

The original contributions presented in this study are included in the article/Supplementary Materials. Further inquiries can be directed to the corresponding authors.

## Supplementary Materials

The following supporting information can be downloaded at https://www.mdpi.com/article/10.3390/vaccines13010025/s1: Figure S1: ^1^H-NMR spectrum of p(PEG_4-5_MA)_a_-*co*-pHMA_b_ in CD_3_OD; Figure S2: ^1^H-NMR spectrum of {p(PEG_4-5_MA)_a_-*co*-pHMA_b_}_X_-*b*-{pBMA_c_-*co*-pDMAEMA_d_-*co*-pPAA_e_}_Y_) in CDCl_3_; Figure S3: Membrane disruption of SM102 LNP alone or in combination with cPMs or PC-cPMs at different pH values—4.5 (lysosome conditions), 5.5 (late endosome conditions), 6.5 (early endosome conditions), and 7.4 (physiological conditions): (a) SM102 LNPs alone; (b) SM102 LNPs (2.1 μg/mL) with different concentrations of cPMs; (c) SM102 LNPs (2.1 μg/mL) with different concentrations of PC-cPMs; Figure S4. Evaluation of intramuscular administration of premixed MC3 LNP-Fluc mRNA (0.3 mg/kg mRNA) and cPM (20 mg/kg) in vivo expression. *n* = 3 per experiment group; Figure S5: Evaluation of intramuscular administration of MC3 LNP-Fluc mRNA (0.3 mg/kg mRNA) and in vivo expression with separate cPM (20 mg/kg) administration in ipsilateral leg: (a) representative images of luciferase expression at the MC3 LNP-Fluc mRNA injection site in the whole body 6 h after administration; (b) total flux (p/s) of luciferase activity calculated using Living imaging software; data are presented as mean ± standard deviation; *n* = 4 per experiment group; Figure S6: Weight change of the mice after the intramuscular administration, i, of MC3 LNP-Fluc mRNA (a) or SM102 LNP-Fluc mRNA and (b) in vivo expression with separate cPM administration. Data are presented as mean ± standard deviation; *n* = 3 per experiment group for MC3 LNP-Fluc mRNA, and *n* = 5 per experiment group for SM102 LNP-Fluc mRNA.
