# Supplementary material for: Development of a Cationic Polymeric Micellar Structure with Endosomal Escape Capability Enables Enhanced Intramuscular Transfection of mRNA-LNPs
Source: Vaccines (Basel). 2024 Dec 30;13(1):25. doi: 10.3390/vaccines13010025 (PMC11768556; doi:10.3390/vaccines13010025)
Supplement: Supplementary file 1 [file vaccines-13-00025-s001.zip › vaccines-3326402-supplementary.pdf]

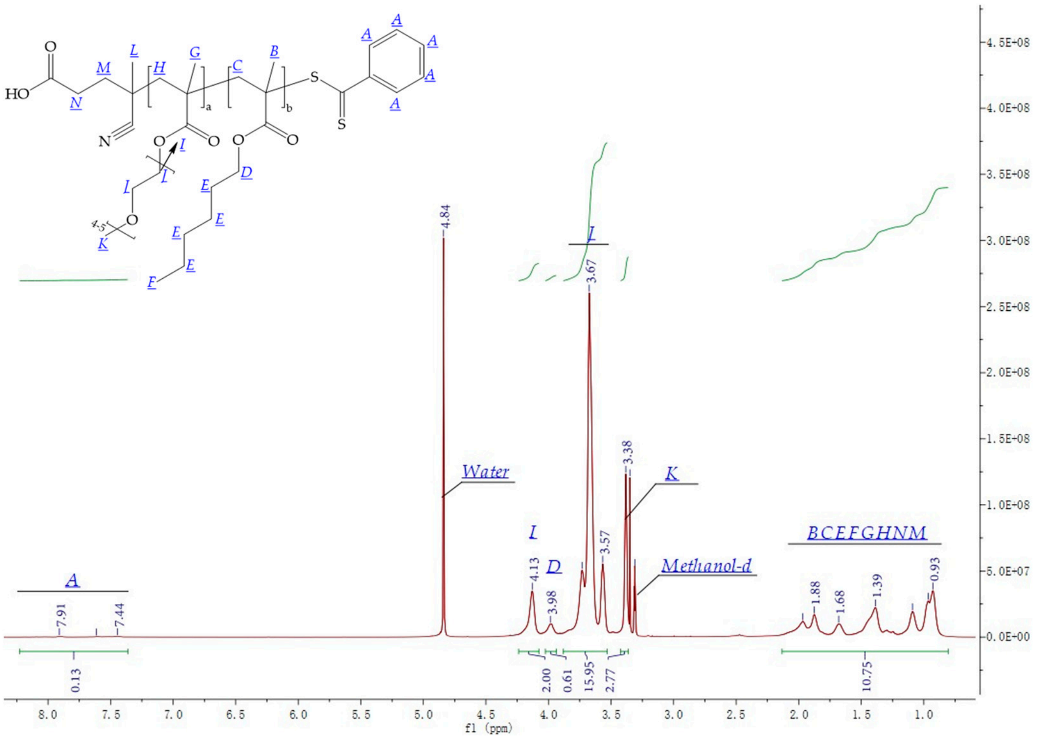

Figure S1. ^{1}H-NMR spectrum of p(PEG$_{4\text{-}5}$MA)$_a$-*co*-pHMA$_b$ in CD$_3$OD

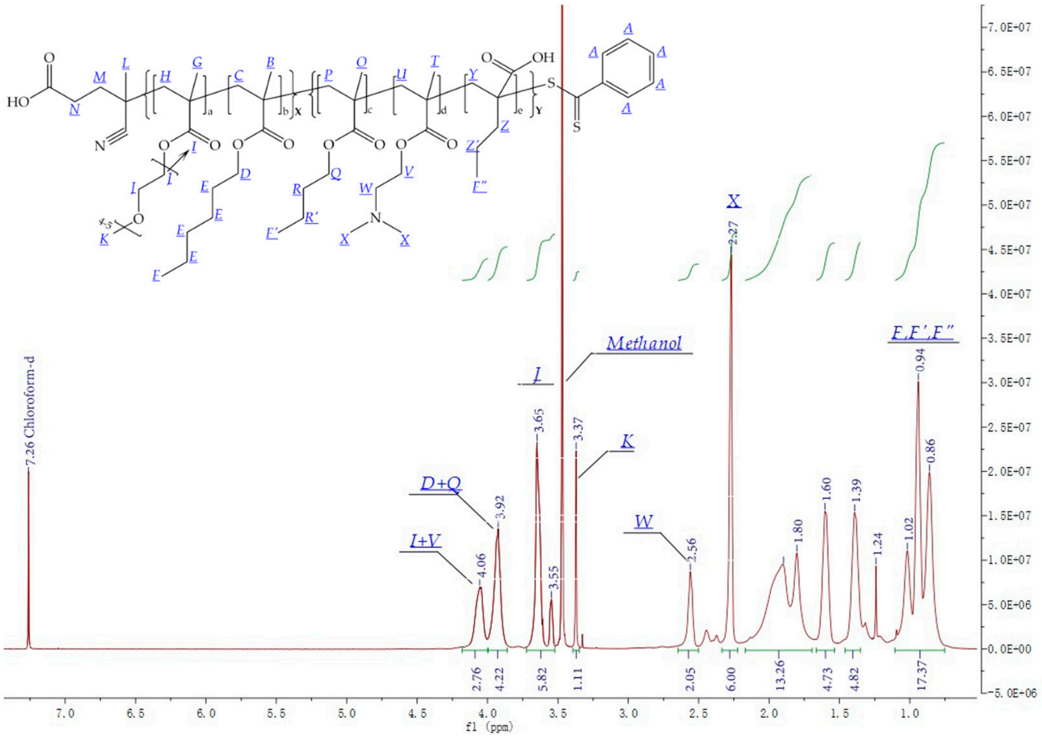

Figure S2. ^{1}H-NMR spectrum of {p(PEG$_{4\text{-}5}$MA)$_a$-*co*-pHMA$_b$}$_X$-*b*-{pBMA$_c$-*co*-pDMAEMA$_d$-*co*-pPAA$_e$}$_Y$) in CDCl$_3$

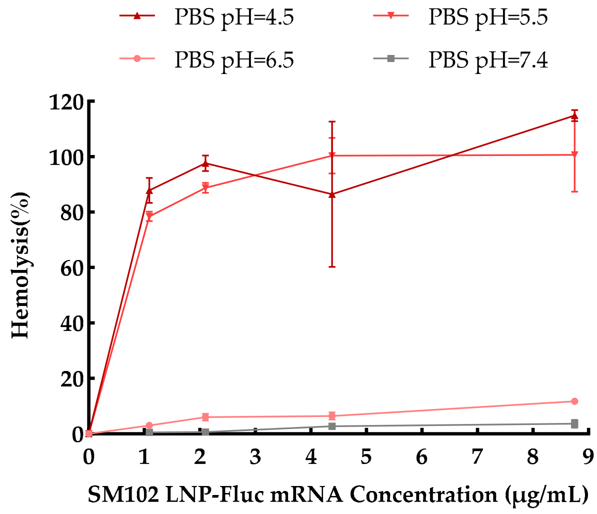

(a)

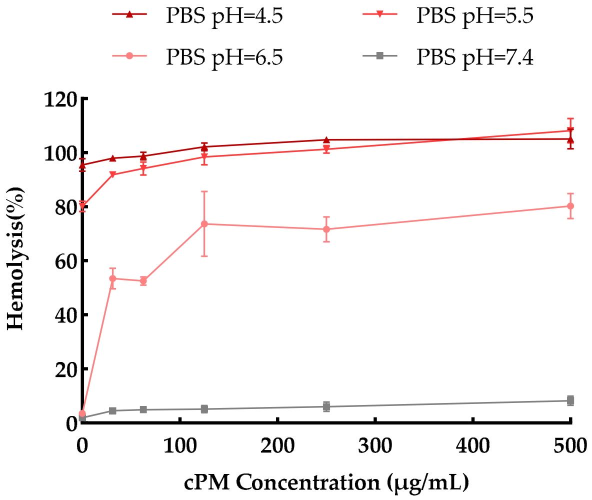

(b)

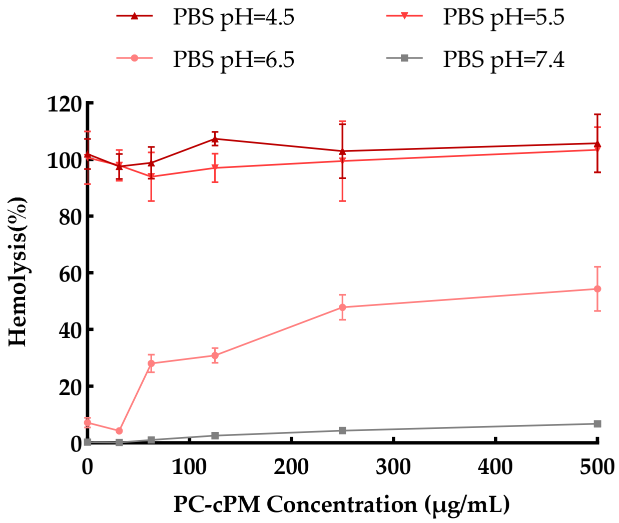

(c)

Figure S3. Membrane disruption of SM102 LNP alone or assisted with cPM or PC-cPM at different pH values 4.5 (lysosome condition), 5.5 (late endosome condition), 6.5 (early endosome condition) and pH 7.4 (physiological condition): (a). SM102 LNP alone; (b). SM102 LNP (2.1 μg/mL) with different concentration of cPM; (c). SM102 LNP (2.1 μg/mL) with different concentration of PC-cPM.

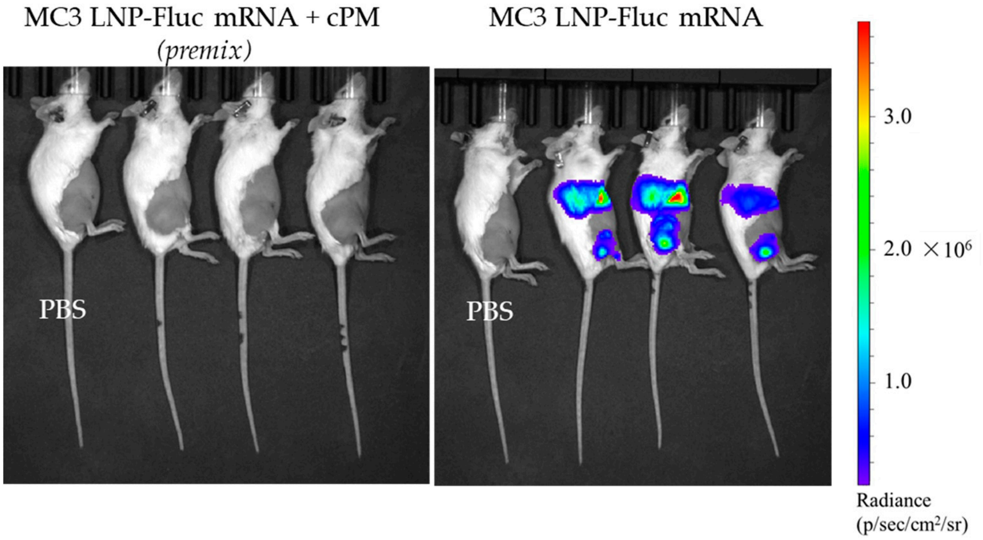

Figure S4. Evaluation of intramuscular administration of premixed MC3 LNP-Fluc mRNA (0.3 mg/kg mRNA) and cPM (20 mg/kg) *in vivo* expression. *n* = 3 per experiment group.

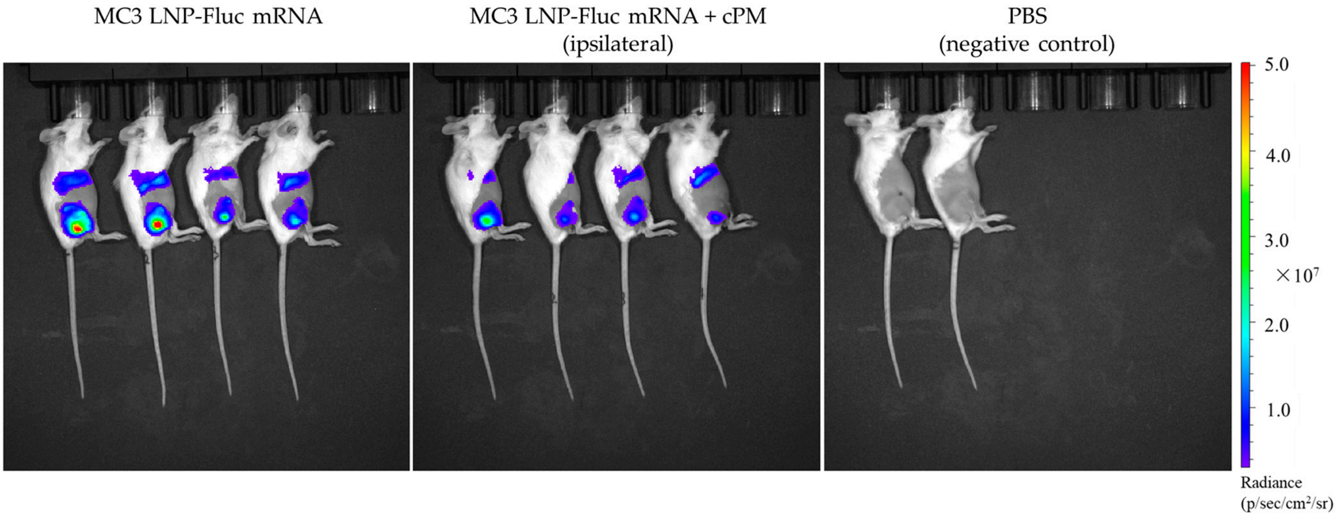

(**a**)

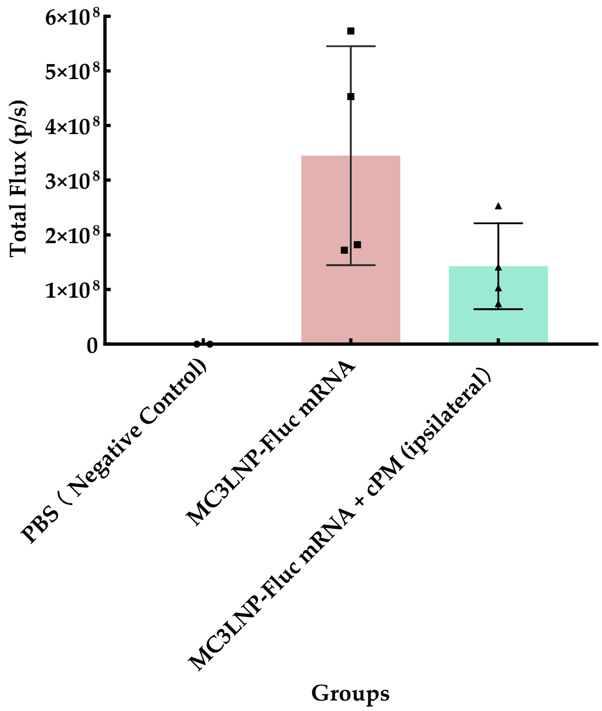

(**b**)

Figure S5. Evaluation of intramuscular administration of MC3 LNP-Fluc mRNA (0.3 mg/kg mRNA) *in vivo* expression with separate cPM (20 mg/kg) administration in ipsilateral leg: (a). Representative images of luciferase expression at the MC3 LNP-Fluc mRNA inject site in the whole body 6 hours after administration; (b). Total flux (p/s) of luciferase activity calculated using Living imaging software; Data is presented as mean ± standard deviation; $n = 4$ per experiment group.

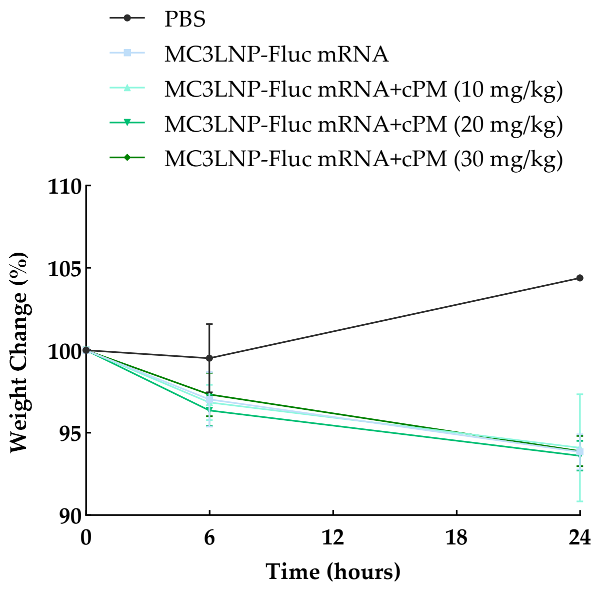

(a)

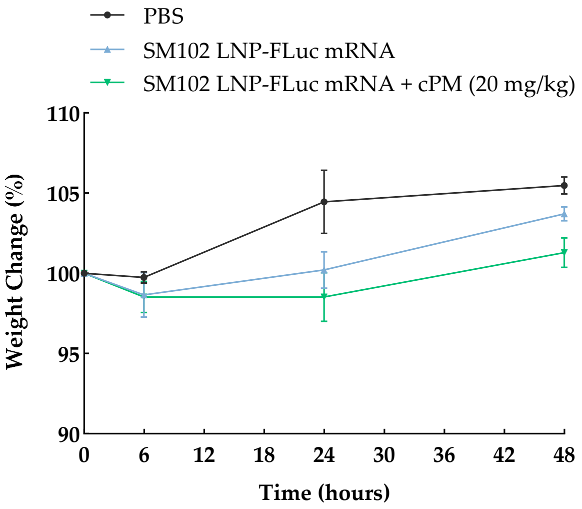

(b)

Figure S6. Weight change of the mice after the intramuscular administration i of MC3 LNP-Fluc mRNA (a) or SM102 LNP-Fluc mRNA (b) in vivo expression with separate cPM administration. Data is presented as mean ± standard deviation; n = 3 per experiment group for MC3 LNP-Fluc mRNA and n = 5 per experiment group for SM102 LNP-Fluc mRNA.
